# Long-Term Effects of Incretin-Based Drugs on Glycemic Control in Permanent Neonatal Diabetes

**DOI:** 10.1210/jcemcr/luae188

**Published:** 2024-10-18

**Authors:** Ayaka Oshiro, Ryoichiro Aotani, Wakako Sakamoto, Takanari Kitazono, Toshiaki Ohkuma

**Affiliations:** Department of Medicine and Clinical Science, Graduate School of Medical Sciences, Kyushu University, Fukuoka 812-8582, Japan; Department of Medicine and Clinical Science, Graduate School of Medical Sciences, Kyushu University, Fukuoka 812-8582, Japan; Department of Medicine and Clinical Science, Graduate School of Medical Sciences, Kyushu University, Fukuoka 812-8582, Japan; Department of Medicine and Clinical Science, Graduate School of Medical Sciences, Kyushu University, Fukuoka 812-8582, Japan; Department of Medicine and Clinical Science, Graduate School of Medical Sciences, Kyushu University, Fukuoka 812-8582, Japan

**Keywords:** permanent neonatal diabetes mellitus, sulfonylurea, dipeptidyl peptidase-4 inhibitor, glucagon-like peptide-1 receptor agonist

## Abstract

Permanent neonatal diabetes mellitus (PNDM) is a genetic disorder, characterized by a decrease in endogenous insulin secretion. Therefore, exogenous insulin supplementation plays a central role in controlling glycemia. Although adding a sulfonylurea can help to discontinue insulin, discontinuation is sometimes difficult when the sulfonylurea is administered at older ages. A 24-year-old woman with longstanding PNDM who had poor glycemic control using insulin (47 U/day) and high-dose glibenclamide (0.6 mg/kg/day), had successfully discontinued insulin after initiating the dipeptidyl peptidase-4 inhibitor sitagliptin (50 mg/day). Additionally, hemoglobin A1c levels decreased by 4.8%. Double dosing of sitagliptin and subsequent switching to the glucagon-like peptide-1 receptor agonist semaglutide (0.25 mg/week followed by 0.5 mg/week) further decreased hemoglobin A1c values, with graded improvements in endogenous insulin secretion. There were no episodes of hypoglycemia during which glibenclamide was titrated down from 0.6 to 0.4 mg/kg/day. Intra- and inter-day glucose variability as assessed by continuous glucose monitoring was also improved. In patients with PNDM, administration and dose escalation of incretin-based drugs, in addition to a high-dose sulfonylurea, could be a useful treatment strategy. This strategy may be helpful for discontinuing insulin, downtitrating sulfonylureas, and subsequent achievement of better glycemic control regarding long-term stability and short-term variability.

## Introduction

Permanent neonatal diabetes mellitus (PNDM) is diagnosed in the first 6 months after birth with a genetic disorder, and it is commonly caused by a pathogenic variant in *KCNJ11*. *KCNJ11* encodes the Kir6.2 subunit of the ATP-sensitive potassium (K_ATP_) channel, which contributes to insulin secretion. PNDM is characterized by a decrease in endogenous insulin secretion. Therefore, exogenous insulin supplementation plays a central role in controlling glycemia. The addition of a sulfonylurea (SU) to insulin therapy is effective in patients with PNDM and may be a treatment option for enabling discontinuation of insulin [1]. However, in some patients, such as those who initiate an SU at older ages, stopping insulin may be difficult, even with administration of high-dose SUs [2].

We previously reported an adult female patient with long-term PNDM (24 years old) and poor glycemic control under the treatment of insulin (47 U/day) and a high-dose SU (0.6 mg/kg/day) [3]. She successfully discontinued insulin after adding sitagliptin, which is a dipeptidyl peptidase (DPP)-4 inhibitor [3]. This finding suggests the effectiveness of activating a non-K_ATP_ channel-mediated pathway to stimulate insulin secretion, not only in type 2 diabetes, but also in PNDM. We report the following clinical course of this patient, during which doubling the dose of sitagliptin and subsequent switching from sitagliptin to semaglutide, a glucagon-like peptide-1 receptor (GLP-1R) agonist, further improved glycemic control regarding short-term intra- and inter-day variability and long-term stability.

## Case Presentation

The patient was diagnosed with type 1 diabetes at 2 months after birth by hyperglycemia (362 mg/dL [20.1 mmol/L]; normal reference range 70-180 mg/dL [3.9-10.0 mmol/L]) and decreased endogenous insulin secretion (serum C-peptide concentration 0.2 ng/mL [0.07 nmol/L]; normal reference range 0.74-3.18 ng/mL [0.25-1.05 nmol/L] and serum immunoreactive insulin concentration < 3 µg/mL; normal reference range 1-18 µg/mL). She was administered insulin treatment.

## Diagnostic Assessment

After having genetic testing at the age of 13 years, she was diagnosed with PNDM (abnormal *KCNJ11* gene; c.754G>T, p.V252L heterozygote), and glibenclamide (0.4 mg/kg/day) was subsequently added to insulin [3]. While the hemoglobin A1c (HbA1c) value temporarily decreased from 10.0% (85.8 mmol/mol; normal reference range 4.9%-6.0% [30.1-42.1 mmol/mol]) to 7.0% (53.0 mmol/mol), it gradually increased to approximately 10.0% (85.8 mmol/mol) to 11.0% (96.7 mmol/mol).

## Treatment

**Figure 1.**
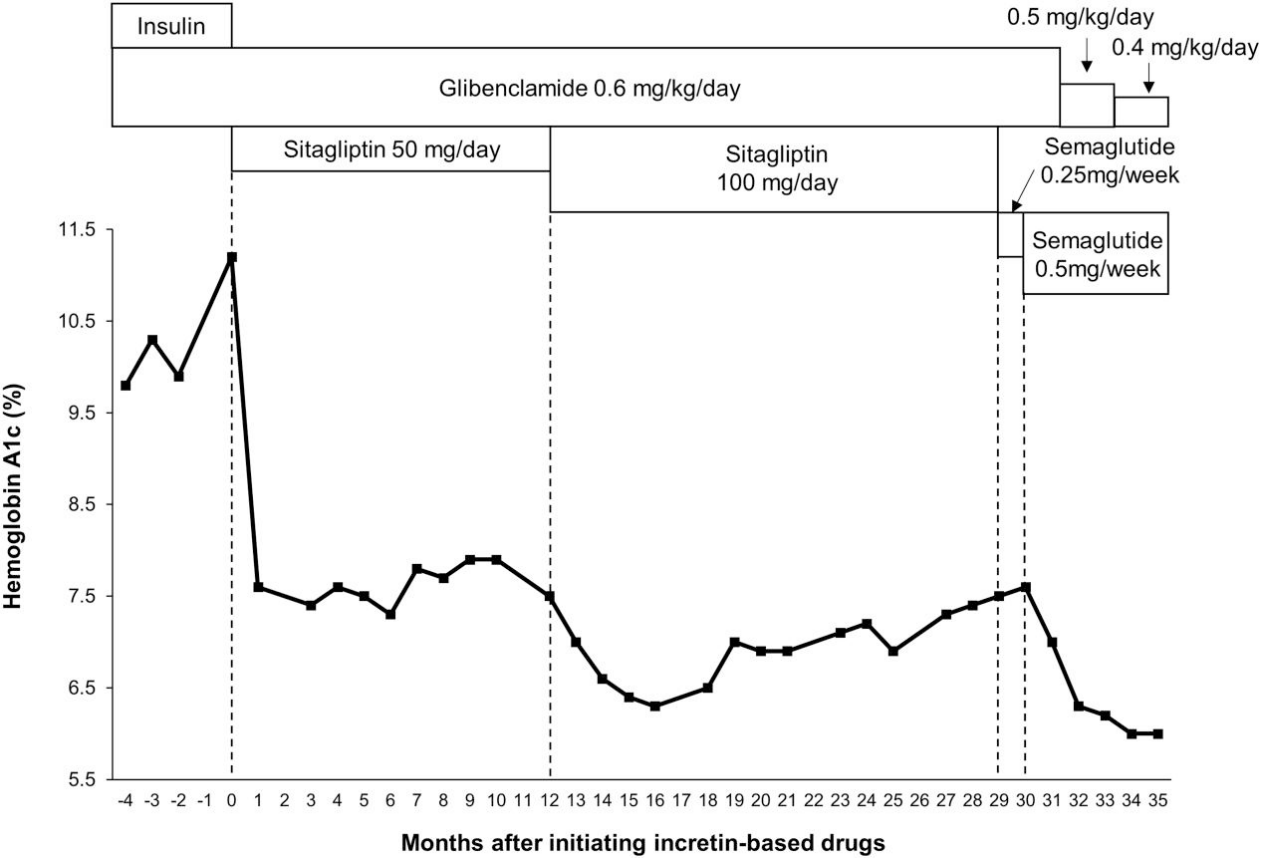
Hemoglobin A1c values before and after administration of incretin-based drugs.

At the age of 24 years, sitagliptin (50 mg/day) was added to insulin (47 U/day; 14 U of basal insulin degludec and 33 U of bolus insulin aspart) and glibenclamide (0.6 mg/kg/day). After the administration of sitagliptin, insulin secretion dramatically improved (urine C-peptide concentration increased from 27.6 to 68.3 μg/day; normal reference range 40-100 μg/day), and she was able to successfully stop taking insulin [3]. The HbA1c value decreased from 11.2% (98.9 mmol/mol) before starting sitagliptin to 7.4% (57.4 mmol/mol) 3 months after starting sitagliptin [3], and it plateaued thereafter at 7.5% (58.5 mmol/mol) (Fig. 1).

Twelve months after adding sitagliptin, the dose was increased from 50 to 100 mg. After doubling the sitagliptin dose, her HbA1c value decreased more than 1%, and then slightly increased and stabilized at approximately 7.0% (53.0 mmol/mol) (Fig. 1). Switching from sitagliptin to subcutaneous semaglutide (0.25 mg/week and then doubled to 0.5 mg/week 1 month later) resulted in additional improvement in glycemic control, without any episode of hypoglycemia.

## Outcome and Follow-Up

**Figure 2.**
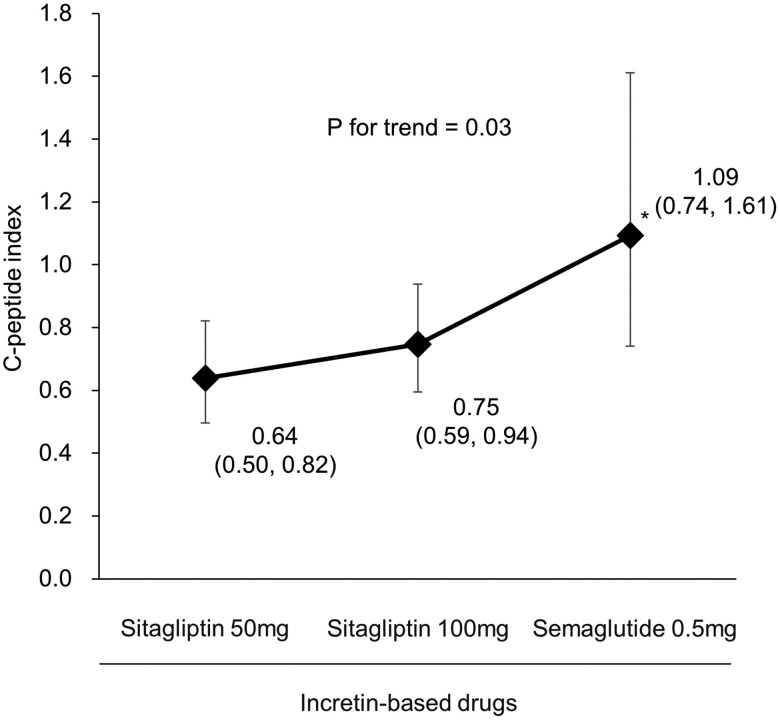
Changes in the C-peptide index during the clinical course. Data in the graph represent geometric means and 95% CI. The C-peptide index was calculated by the serum C-peptide concentration (ng/dL)/serum glucose concentration (mg/dL) × 100, under a nonfasting state. The C-peptide index was log-transformed for statistical analyses. Differences between subgroups were tested by the unpaired *t* test, and the linear trend across groups was tested by a linear regression model. *P < .05 vs sitagliptin 50 mg. Dosages of glibenclamide were the same as those described in Fig. 1.

**Figure 3.**
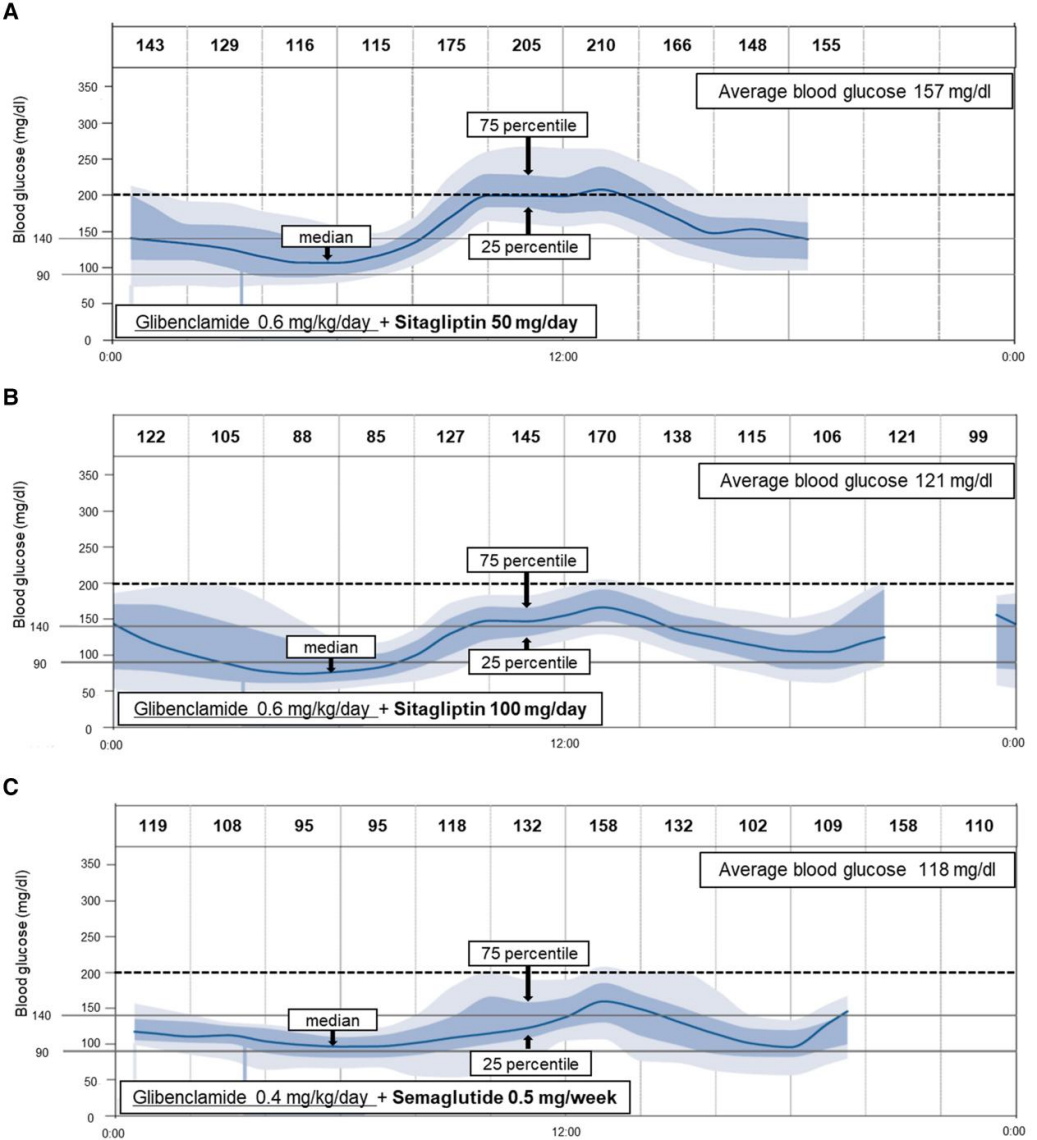
Glucose concentrations assessed by continuous glucose monitoring before (A) and after (B) doubling the dose of sitagliptin, and (C) switching to semaglutide.

The HbA1c value at 6 months after initiating semaglutide was 6.1% (43.2 mmol/mol), during which glibenclamide was titrated down from 0.6 mg/kg/day to 0.4 mg/kg/day. Endogenous insulin secretion was also improved (Fig. 2). The C-peptide index (serum C-peptide concentration [ng/dL]/serum glucose concentration [mg/dL] × 100) [4] increased after doubling the dose of sitagliptin and further increased after switching to semaglutide despite downtitrating the SU dosage. The average daily glucose concentration decreased from 157 to 121 mg/dL (from 8.72 to 6.72 mmol/L). Glucose concentrations evaluated by intermittently scanned continuous glucose monitoring (isCGM) also showed a marked improvement in their fluctuation during the daytime, compared with before and after doubling the dose of sitagliptin (Fig. 3A and 3B). In addition, switching to semaglutide from sitagliptin markedly decreased day-to-day glycemic variability, particularly during the nocturnal period (Fig. 3B and 3C). Over the course of treatment, there were no changes in diet or exercise habits. Body weight remained stable at 54 kg, followed by a reduction to 52 kg after the dose escalation of semaglutide to 0.5 mg/week. Additionally, no diabetic vascular complications were observed.

## Discussion

We experienced a patient with PNDM who had successfully discontinued insulin after initiating sitagliptin, but did not have sufficient glycemic control. Increasing the dosage of sitagliptin from 50 to 100 mg and subsequent transition to semaglutide safely achieved the glycemic target of an HbA1c value of < 7% (53.0 mmol/mol), with a remarkable improvement in insulin secretion capacity. During the clinical course, glibenclamide was downtitrated from 0.6 mg/kg/day to 0.4 mg/kg/day and no episode of severe hypoglycemia was observed. These findings indicated long-term efficacy and safety of incretin-based drugs on glycemic control in our patient with PNDM.

Several previous studies in patients with type 2 diabetes have shown a dose-dependent improvement in glycemic levels by the DPP-4 inhibitor sitagliptin. A study conducted in the UK reported that HbA1c values decreased by 0.43% and 0.54% from baseline in participants with sitagliptin 50 mg/day and 100 mg/day, respectively [5]. Another report of patients with Japanese type 2 diabetes showed that sitagliptin led to a dose-related improvement in beta-cell function, in addition to a decrease in HbA1c values [6]. DPP-4 inhibitors also showed dose-dependent increases in beta-cell mass in a mouse model of type 2 diabetes, leading to the normalization of beta-cell mass and an improvement in islet architecture [7]. The findings in our patient support these previous findings [5-7] of dose-dependent improvement in glycemic control and insulin secretion capacity by sitagliptin observed in type 2 diabetes, and further show its effect on PNDM.

The glycemic lowering effect of a GLP-1R agonist in PNDM was previously reported in a 40-year-old Danish man [8], who successfully discontinued insulin after initiating liraglutide. However, whether an improvement in beta-cell function observed in type 2 diabetes after switching from a DPP-4 inhibitor to a GLP-1R agonist [9] is also found in PNDM remains unclear. The present patient experienced substantial improvement in insulin secretion capacity, as evaluated by the C-peptide index after the transition to semaglutide (Fig. 2). This finding suggests the usefulness of a GLP-1R agonist in PNDM characterized by impaired insulin secretion as the main pathophysiology, which is different from that of type 2 diabetes consisting of insulin resistance and impaired insulin secretion.

In our patient, a dose-dependent effect of sitagliptin and an effect of transition from sitagliptin to semaglutide were clearly found, not only on HbA1c values, which reflect long-term glycemic control, but also on short-term intra- and inter-day glucose variability, which was assessed by isCGM (Fig. 3). To the best of our knowledge, this is the first case report to show the glucose-lowering effect of incretin-based drugs added to high-dose glibenclamide using long-term (HbA1c) and short-term (isCGM) measures in a patient with PNDM. There is an association between CGM-derived time-in-range and microvascular complications, and this association is independent of long-term glycemic measurement of HbA1c [10]. Therefore, addition and subsequent dose escalation of incretin-based drugs could be useful for glycemic control and the following prevention of diabetic complications.

Switching from sitagliptin to semaglutide enabled us to reduce the dosage of a high-dose SU from 0.6 to 0.4 mg/kg/day and also to safely achieve glycemic control without any episode of hypoglycemia. In patients with PNDM and a high-dose SU, 10.7% of patients were reported to experience hypoglycemia once weekly or more [11]. There is evidence on the effect of hypoglycemia on cardiovascular disease [12] and cognitive decline [13]. Therefore, downtitrating a high-dose SU prescription, which has a high potential to cause hypoglycemia, would be greatly beneficial for the long-term prognosis. Taken together, these findings suggest the importance of dual use of insulin secretagogues, which activate K_ATP_ channel- and non-K_ATP_ channel-mediated pathways, in PNDM.

In conclusion, administration and dose escalation of incretin-based drugs, in addition to a high-dose SU, could be an effective treatment strategy in PNDM. This strategy may be useful for not only discontinuing insulin, but also for decreasing SUs, and subsequent achievement of better glycemic control with minimizing the risk of hypoglycemia.

## Learning Points

- Permanent neonatal diabetes mellitus (PNDM) is a genetic disorder characterized by a decrease in endogenous insulin secretion, suggesting that exogenous insulin supplementation plays a central role in controlling glycemia.
- Although adding a sulfonylurea (SU) can help to discontinue insulin, discontinuation is sometimes difficult when a SU is administered at older ages, even with a high-dose SU prescription.
- Incretin-based drug therapy added to a high-dose SU markedly improved glycemic control and enabled discontinuation of insulin, with remarkable improvement in endogenous insulin secretion.
- During the clinical course, the SU was downtitrated and no episode of severe hypoglycemia was observed.
- Dual use of insulin secretagogues, which activate K_ATP_ channel- and non-K_ATP_ channel-mediated pathways, could be an effective treatment strategy in PNDM.

## Data Availability

All datasets generated and analyzed during the current manuscript are not publicly available but are available from the corresponding author on reasonable request.

## Abbreviations

DPP: dipeptidyl peptidase
GLP-1R: glucagon-like peptide-1 receptor
HbA1c: glycated hemoglobin
isCGM: intermittently scanned continuous glucose monitoring
PNDM: Permanent neonatal diabetes mellitus
SU: sulfonylurea
